# N-Acetylcysteine: A Review of Clinical Usefulness (an Old Drug with New Tricks)

**DOI:** 10.1155/2021/9949453

**Published:** 2021-06-09

**Authors:** Gerry K. Schwalfenberg

**Affiliations:** Department of Family Medicine, University of Alberta, No. 301, 9509-156 Street, Edmonton T5P 4J5, AB, Canada

## Abstract

**Objective:**

To review the clinical usefulness of N-acetylcysteine (NAC) as treatment or adjunctive therapy in a number of medical conditions. Use in Tylenol overdose, cystic fibrosis, and chronic obstructive lung disease has been well documented, but there is emerging evidence many other conditions would benefit from this safe, simple, and inexpensive intervention. *Quality of Evidence*. PubMed, several books, and conference proceedings were searched for articles on NAC and health conditions listed above reviewing supportive evidence. This study uses a traditional integrated review format, and clinically relevant information is assessed using the American Family Physician Evidence-Based Medicine Toolkit. A table summarizing the potential mechanisms of action for N-acetylcysteine in these conditions is presented. *Main Message.* N-acetylcysteine may be useful as an adjuvant in treating various medical conditions, especially chronic diseases. These conditions include polycystic ovary disease, male infertility, sleep apnea, acquired immune deficiency syndrome, influenza, parkinsonism, multiple sclerosis, peripheral neuropathy, stroke outcomes, diabetic neuropathy, Crohn's disease, ulcerative colitis, schizophrenia, bipolar illness, and obsessive compulsive disorder; it can also be useful as a chelator for heavy metals and nanoparticles. There are also a number of other conditions that may show benefit; however, the evidence is not as robust.

**Conclusion:**

The use of N-acetylcysteine should be considered in a number of conditions as our population ages and levels of glutathione drop. Supplementation may contribute to reducing morbidity and mortality in some chronic conditions as outlined in the article.

## 1. Introduction

**Figure 1:**
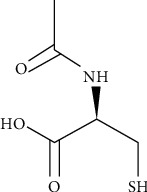
N-acetylcysteine formula. N-acetylcysteine may otherwise be called N-acetyl-L-cysteine, NAC, or NALC. Trade names for N-acetylcysteine are Mucomyst, Fluimucil, and Acetadote. The molecular formula is C5H9NO3S. Its chemical structure is shown (source is https://en.wikipedia.org/wiki/Acetylcysteine).

N-acetylcysteine (NAC) is a sulfhydryl-containing compound, with mucolytic properties, originally patented in 1960, and its use in medicine was first reported in 1967 [1]. Its chemical structure and nomenclature are depicted in Figure 1. Clinically it has been used in cystic fibrosis since 1969 [2]. Since then, NAC use has been expanded to acetaminophen overdose and chronic obstructive lung disease and its role is ever expanding clinically.

Cysteine is found naturally in meat, fish, grains, dairy, soybean, and egg products [3]. As a nutritional supplement, NAC is found in small amounts naturally in some fruits and vegetables [4].

The properties of NAC include enhancing glutathione S-transferase activity, repleting glutathione, scavenging free radicals, and stabilizing protein structures by crosslinking cysteine disulfide molecules along with its antioxidant, anti-inflammatory, and mucolytic properties. A more complete list of mechanisms of action is given in Table 1.

The bioavailability of oral NAC in humans is between 4 and 9.1% in one study [20] and between 6 and 10% in another [21]; thus, studies using less than 1200 mg per day may show no significant benefit. The half-life of NAC is 6.25 hours, and clearance is both renal and nonrenal, with side effects of nausea, vomiting, and diarrhea [22].

Some have suggested that cysteine deficiency as we age is responsible for loss of youth and loss of health and quality of life and contributes to sarcopenia, especially since cysteine consumption is considered suboptimal [23].

There have been several systematic reviews of NAC in the literature over the past decade looking at various clinical trials in psychiatry and neurology [24], metabolic disease [25], pulmonary disease [26], infectious diseases including possible use in acute respiratory syndromes like corona virus 2 (SARS-CoV-2) [27], and infertility [28] and as chelator for metal toxicity [14].

## 2. Methods

This review of NAC and its relevance in clinical practice was prepared by assessing medical and scientific literature available on MEDLINE and PubMed as well as by reviewing books and conference proceeding. A traditional integrated review format was used in this review where there is limited primary study, thus endeavoring to provide a useful overview of the literature as it pertains to clinical uses for the clinician. The literature reviewed was from the past forty years with emphasis on human studies with preference for randomized control trial (RCT) studies or meta-analysis. Preclinical studies in cell cultures or animal studies were added when relevant. The terms searched for were N-acetylcysteine, NAC, acetaminophen, cystic fibrosis, chronic obstructive lung disease, asthma, bronchitis, bronchiectasis, idiopathic pulmonary fibrosis, sarcopenia, Parkinson's disease, dementia, multiple sclerosis, acquired immune deficiency syndrome (AIDS), tuberculosis, influenza, SARS-CoV-2, glutamate, glutathione, contrast nephropathy, schizophrenia, autism, obsessive compulsive disorder, polycystic ovary disease, male infertility, sleep apnea, cancer, hypertension, ulcerative colitis, and others.

The American Family Physician Evidence-Based Medicine Toolkit was used to determine the level of evidence where clinically relevant information was assessed [29]. Assessing level of evidence is important for clinicians and this tool kit was used to provide a sense of the strength of recommendations based on a body of evidence.

**Figure 2:**
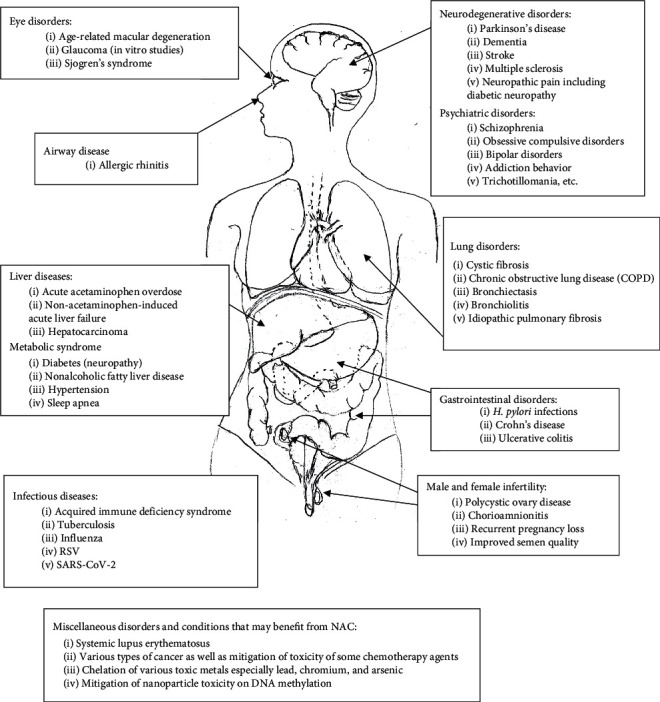
A graphic representation of the clinical uses of N-acetylcysteine (NAC) in various organ systems, as treatment or adjuvant therapy.

An overview of the use of NAC in clinically relevant disorders is depicted in Figure 2.

### 2.1. N-Acetylcysteine in Lung Disorders

Studies on lung disorders in humans is an expanding topic with early beginnings using inhaled Mucomyst for cystic fibrosis. Oral NAC is now being used for conditions like chronic obstructive lung disease and other conditions as outlined below.

### 2.2. Cystic Fibrosis (LOE = A)

Cystic fibroses (CF) is a multiorgan genetic recessive disorder that affects 1/2500 births and is characterized by hyperviscoelastic sputum, neutrophilic inflammation, and infection.

High-dose oral NAC was used safely in modulating inflammation, improving glutathione levels, and decreasing elastase activity [30]. A recent study showed that lung function was maintained with oral NAC over 24 weeks, while there was deterioration on placebo; however, other markers of inflammation did not change [31]. A 1999 Cochrane Review of nebulized NAC did not show significant benefit and nebulized NAC had a very bad taste and smell. Oral NAC studies were small and only showed minimal benefit [32]. However, a recent study on nebulized NAC did show protection of lung function in children [33]. Distal intestinal obstruction found in CF patients may benefit from a nonsurgical alternative using NAC although further studies are warranted [34].

### 2.3. Chronic Obstructive Lung Disease and Chronic Bronchitis (LOE = A,C)

As early as 1985, it was reported that, in chronic obstructive lung disease (COPD), polymorphonuclear leukocyte oxidative damage was reduced with the use of NAC in a controlled in vitro cell model [35]. More recently, a meta-analysis looked at chronic bronchitis and COPD treated with NAC, and there were significantly fewer exacerbations in the treated group than in the placebo group. For those with documented airway obstruction, recommendations are to take 1200 mg/day, as a preventative [36].

A quantitative systematic review using NAC in chronic bronchitis showed significant benefit in preventing exacerbation and the number needed to treat (NNT) to achieve benefit was 5.8 [37]. Recommendations as add-on therapy for COPD and chronic bronchitis is considered a reasonable approach [38].

### 2.4. Asthma and Allergy (LOE = B)

Studies at present do not support the use of NAC in acute asthma attacks because of lack of improvement of cough, wheezing, dyspnea, sputum expectoration, or night sleep [39]. However, in animal models, steroid-resistant acute exacerbation of asthma did benefit from NAC [40]. To date, there have been no long-term studies using NAC in prevention of recurrent asthma attacks by reducing inflammation and mucous plugging. NAC has been shown to reduce the allergen-induced nasal inflammatory cascade in allergic rhinitis in animal models. [41]. Topical application of NAC prior to ragweed exposure resulted in attenuation of the late phase allergic response mediated nasal symptoms [42].

### 2.5. Bronchiectasis (LOE = A)

There is in vitro evidence that biofilms are disrupted and prevented with use of NAC potentially reducing infection [43]. A recent long-term RCT study using NAC 600 mg bid resulted in fewer exacerbations (RR 0.41), less 24-hour mucous production, and a significant improvement in quality of life [44].

### 2.6. Bronchiolitis (LOE = A)

Nebulized NAC used in children was found to be effective by reducing the clinical severity score within 3–5 days and resulted in earlier discharge [45]. Further studies are in progress.

### 2.7. Idiopathic Pulmonary Fibrosis (LOE = A)

Idiopathic pulmonary fibrosis is a fatal lung disease with limited options and poor prognosis. In 2011, the use of three medications Azathioprine, Prednisone, and NAC in combination was halted because of increased hospitalizations and deaths. The evidence against the use of these three medications together was strong [46]. Evidence has been mixed over the years but more recently a systematic review and meta-analyses have provided more clarity in the clinical use of NAC in pulmonary fibrosis showing benefit in improving oxygenation and reduced the decline in lung function; however, complications and mortality have remained similar [47].

## 3. Liver and Bowel Diseases

### 3.1. Acetaminophen Overdose (LOE = A)

The usefulness of NAC to prevent acute liver failure has been well established if used according to well-established intravenous dose protocols within 8 hours of ingestion [48]. NAC as an antidote for acetaminophen-induced (AI) liver damage was first written about in 1977 and was more widely accepted by the mid 1980s [49]. The reactive metabolite of acetaminophen is detoxified by glutathione. NAC restores glutathione levels, thus preventing irreversible damage.

### 3.2. Non-Acetaminophen-Induced Acute Liver Failure (LOE = A)

The etiology of non-acetaminophen-induced acute liver failure (NAI-ALF) may include agents like viruses, drugs, toxins, herbal and traditional medications, and autoimmunity. Treatment includes removing the offending agent or specific treatments for the agent. NAC has been used in mushroom poisoning [50], herbicide (Paraquat) poisoning [51], chloroform poisoning [52], and protecting against polychlorinated biphenyls (PCB) induced steatosis [53] and other poisonings. NAC has been used because of its antioxidant, anti-inflammatory, and vasodilating effects as seen in acetaminophen damage [54]. The use of NAC in NAI-ALF reduced mortality and average length of stay and improved survival [55, 56]. A meta-analysis of prospective clinical trials reviewing NAC-treated and placebo-treated groups showed NAC to be safe and prolonged the survival of patients with native livers without transplantation but did not improve overall survival. [57].

### 3.3. Hepatocarcinoma (LOE = B)

Liver cancer, most commonly known as hepatocarcinoma, is a common malignancy, and treatment of this cancer with interferon-alpha 2A (IFN-*α*) has a relatively poor response rate of about 30%. NAC acts synergistically to improve the efficacy of the drug by decreasing tumor viability, increasing apoptosis, and decreasing expression of nuclear factor kappa-light-chain-enhancer of activated B cells (NF-kB). Inactivating the pathway of initiation, promotion, and progression of tumors by NF-kB can be done by using NAC as an adjuvant to IFN *α* [58].

### 3.4. Crohn's Disease (LOE = A)

Crohn's disease is characterized by marked systemic oxidative stress even in those in clinical remission [59]. In a double-blind RCT (*N* = 168), the relapse rate of those on NAC 400 mg twice daily was significantly reduced compared to placebo while tapering off prednisolone [60].

### 3.5. Ulcerative Colitis (LOE = A)

The use of antioxidant therapy in inflammatory bowel disease has been suggested in the past [61]. In an RCT, using 400 mg of NAC twice a day in ulcerative colitis patients who were on prednisolone taper, the relapse rate was significantly less in the treatment group. The endoscopic relapse rate, serum level of high-sensitivity C-reactive protein (hs-CRP), and fecal calprotectin level were all lower in the treatment group [62].

### 3.6. Systemic Lupus Erythematosus (LOE = A)

There is depletion of glutathione in patients with systemic lupus erythematosus as well as T-cell dysfunction. NAC replenishes glutathione and as an antioxidant in and of itself is able to inhibit mechanistic target of rapamycin (mTOR) in vitro [62]. A double-blind RCT pilot study using 2.4 gm of NAC daily safely and significantly improved lupus disease activity [63].

## 4. Metabolic Syndrome including Nonalcoholic Fatty Liver Disease, Diabetes, and Polycystic Ovary

### 4.1. Nonalcoholic Fatty Liver Disease (LOE = B)

There is evidence that NAC may block hepatic lipid accumulation and provide therapeutic benefit against metabolic complications found in nonalcoholic fatty liver disease (NAFLD). This is primarily due to the antioxidant effects and attenuation of lipid peroxidation [64]. This is supported by most preclinical studies and a few clinical studies, and there is an urgent need for larger clinical studies. This disorder affects up to 25% of the population, a condition which may lead to significant pathology such as fibrosis of the liver.

### 4.2. Diabetes

Animal studies show that NAC may inhibit hepatic steatosis and development of glucose intolerance and improve lipid profiles [65, 66]. In type 2 diabetic humans, there does not appear to be any benefit in improving glucose tolerance or *ß*-cell function with the addition of NAC in short-term trials (2 weeks) [67]. Long-term trials are warranted.

### 4.3. Diabetic Neuropathy, Retinopathy, and Nephropathy (LOE = A)

NAC has been shown to benefit diabetic peripheral neuropathy in animals [68]. Likewise, animal studies investigating retinopathy [69] and diabetic nephropathy [70] show promising results. Recently, a study in humans has shown significant benefit in painful peripheral neuropathy [71]. Patients with painful neuropathy improved over 8 weeks with the use of NAC (600 mg bid) as adjuvant therapy to pregabalin. There was a >50% reduction in pain score compared to placebo.

### 4.4. Polycystic Ovary Disease, Chorioamnionitis, and Recurrent Pregnancy Loss (LOE = A, B)

Treatment with NAC may improve insulin sensitivity in women with polycystic ovary disease [72]. A systematic review of RCTs using NAC as a supplement in polycystic ovary disease resulted in improved fertility, ovulation, and odds of having a live birth although these results were not as robust as studies using Metformin [73]. The dosage in studies varied from 1200 mg/day to 1800 mg/day. Chorioamnionitis, a devastating infection with increased risk of cerebral palsy and other neurological sequalae, may benefit from intravenous NAC (both antenatally and postnatally) by reducing neuroinflammation [74]. The use of NAC as an adjunct in recurrent pregnancy loss has been shown to improve the take-home-baby rate as compared to folic acid alone [75].

### 4.5. Male Fertility (LOE = A)

In idiopathic male infertility, an RCT using NAC showed improvement of oxidative status along with semen quality (improved motility, viscosity, and volume) [76]. The dosage was 600 mg twice daily. In another RCT using selenium and NAC together, there was significant improvement in semen quality [77].

### 4.6. Hypertension (LOE = B)

Cysteine-rich diets such as the dietary approaches to stop hypertension (DASH) diet improve insulin resistance, decrease oxidative stress, lower advanced glycation end products, increase the storage form of glutathione, and modulate nitric oxide and other vasoactive molecules, thus lowering blood pressure [3]. However, NAC as add-on therapy in nondiabetic patients with chronic kidney disease who were on renin-angiotensin system blockade medication had no effect on blood pressure [78]. N-acetylcysteine 1800 mg daily over 1 month significantly lowered homocysteine levels and systolic and diastolic blood pressure in middle-aged men who were hyperlipidemic [79]. The combination of NAC and L-arginine in diabetic patients results in increased nitric oxide production and improves systolic blood pressure [19].

#### 4.6.1. Pulmonary Hypertension

There is some evidence that NAC may inhibit the development of pulmonary artery hypertension (PAH) or significantly reduce pulmonary vascular remodeling in animal models [80, 81]. Previous information was contrary to this [82] and it was thought that PAH was a side effect of NAC.

### 4.7. Chemotherapy

Cyclophosphamide is known to have cardiotoxicity which may be mitigated by NAC [83] and cyclophosphamide may cause hemorrhagic cystitis which may be prevented with NAC [84]. Acute kidney damage as seen with cisplatin may be reduced as seen in animal studies [85]. In pediatric cancers studied in mouse models, the use of NAC blocks the side effects of cisplatin but may interfere with its effectiveness if used together. The use of NAC needs to be separated from chemotherapy by at least 4 hours [86].

### 4.8. Breast Cancer, Prostate Cancer, Lung Cancer, Glioblastoma, and Chronic Lymphocytic Leukemia

There are many studies looking at the effect of NAC on cancer cells. By changing the environment in and surrounding the cells, there appears to be benefit in both human and cell studies.

There has been a human pilot study determining that NAC has antiproliferative effects on breast cancer [87]. NAC markedly reduces monocarboxylate transporter 4 (MCT4) transporter proteins from being utilized to import energy as lactate to cancer cells. MCT4 is considered a marker of aggressive cancer behaviour with poor overall survival.

In a prostate cancer cell study, NAC suppressed prostate cancer cell growth [88] and prevented adhesion and invasion to remote locations [88].

Cell studies in lung cancer show that NAC has the ability to detoxify chemicals as a precursor of reduced glutathione, scavenging of radicals, and protection from DNA damage. However, there is some caution since vitamin E and NAC in a combination study showed increased tumor cell proliferation by reducing reactive oxygen species and reducing p53 expression (which increases tumor growth) in mouse and human lung tumor cells [89], whereas NAC and a major tea polyphenol epigallocatechin-3-gallate (EGCG) form an adduct which may enhance EGCG cell killing of cancer cells [90].

Imatinib is an agent successfully used in chronic lymphocytic leukemia and NAC enhances its effectiveness in animal models by increasing the production of nitric oxide [91].

In glioblastoma cell studies, NAC has the remarkable ability to inhibit tumor growth and cell proliferation [92]. Further studies regarding NAC and its role in cancer therapy are needed.

### 4.9. Sleep Apnea (LOE = B)

Sleep apnea has become a significant problem that may lead to hypertension, stroke, and various cardiovascular ailments. This condition is considered a proinflammatory vascular risk factor and NAC has been thought to improve this [93]. There are small clinical RCT trials that have shown benefit [94, 95].

## 5. Infectious Disease

### 5.1. Overview

NAC may strengthen immune defence by increasing the glutathione pool in leukocytes, thus decreasing the likelihood of infections [96].

### 5.2. Acquired Immune Deficiency Syndrome (LOE = A)

In a double-blind placebo-controlled trial using 800 mg of NAC, there was a reduction in the decline of the CD4 count (number of a type of white blood cell) seen in the placebo group and tumor necrosis factor-alpha (TNF-alpha) levels were also reduced [97]. Glutathione levels are improved with NAC, inhibiting actions of inflammatory cytokines and slowing cachexia and wasting [98].

### 5.3. Tuberculosis (LOE = A)

Clinically, NAC as add-on therapy for tuberculosis (TB) treatment significantly improved clearing of infiltration and reduction in cavity size radiologically and brought about faster sputum negativity [99]. In hospitalized TB patients, NAC was associated with a significant reduction in all-cause mortality within 90 days of admission [100].

### 5.4. Influenza, Respiratory Syncytial Virus, and SARS-CoV-2 (LOE = A, B)

A double-blind RCT in which 262 patients were given 600 mg of NAC or placebo for 6 months (during the winter) was conducted to determine the effect of long-term treatment on influenza. This study showed that only 25% of virus-infected patients in the NAC group were symptomatic compared to 79% of patients in the placebo group. There was a significant decrease in influenza-like episodes, severity, and length of time confined to bed and sharp reduction of both local and systemic symptoms in the NAC group. Replication of seasonal human influenza A viruses is inhibited by NAC along with inhibition of virus-induced proinflammatory responses [101].

In cell cultures, inhibition of mucin synthesis and reduction of proinflammatory mediators are seen in alveolar type II epithelial cells which are infected with respiratory syncytial virus (RSV) and influenza A and B viruses [102].

Thiols block the angiotensin-converting enzyme 2, thereby inhibiting penetration of SARS-CoV-2 into cells. In [103], recent reviews of NAC and SARS-CoV-2 (COVID-19) show promise as an agent to modify the immune response and possibly reduce morbidity and mortality [27, 104]. In a case report about one patient with severe COVID-19, NAC demonstrated significant benefit [105]. There are several studies that are in progress.

### 5.5. *H. pylori* (LOE = B)

The additive effect of NAC in the usual treatment of *Heliobacter pylori* (*H. pylori*) appears to improve eradication rates by reducing mucus [106, 107]. It is suggested that NAC may prevent gastritis induced by the organism [108]. In a Cochrane review, the evidence from most studies is weak; however, further studies are warranted [109].

## 6. Neurodegenerative Disorders

### 6.1. Overview

There is evidence that NAC may be protective for neurodegenerative disorders like Parkinson's disease, Alzheimer's disease, neuropathic pain, stroke, and multiple sclerosis (MS). As a glutathione precursor with antioxidant and anti-inflammatory properties, it may be helpful as an adjuvant for these conditions [24, 110].

### 6.2. Parkinson's Disease (LOE = B)

Dopamine may trigger apoptosis in neuronal cell cultures, which may initiate inappropriate loss of nigral cells in Parkinson's disease. Thiols containing compounds like NAC are markedly protective by inhibiting dopamine-induced cell death in cell cultures [111]. A clinical study using NAC both as a weekly intravenous infusion and 500 mg orally twice a day over three months significantly improved Parkinson symptoms and increased dopamine binding in the brain warranting further study [112].

### 6.3. Dementia (LOE = B)

Animal studies have shown significant promise improving cognitive function even though beta-amyloid pathology was unchanged [113]. There is some evidence that NAC as an adjuvant may slow the progression of dementia; however, this effect was seen clinically in a nutraceutical containing several ingredients including NAC [114].

### 6.4. Neuropathic Pain (LOE = A, B)

Matrix metalloproteinases (MMPs) are one of the key components inducing neural inflammation and facilitating inflammatory cytokine maturation. N-acetylcysteine by inhibiting MMP significantly attenuates neuropathic pain in animal studies [115].

As seen above, there is benefit with NAC in diabetic neuropathy, and there is evidence that NAC at 1200 or 2400 mg daily may reduce the incidence and severity of paclitaxel-induced peripheral neuropathy in chemotherapy treatment [116].

### 6.5. Stroke (LOE = A)

Acrolein-mediated damage after stroke has been implicated in the size of strokes in animal studies and NAC has been shown to reduce the size of the infarct [117]. In a recent randomized double-blind placebo-controlled trial using NAC 4 grams four times a day for 72 hours at the onset (within 24 hours) of an ischemic stroke, the follow-up National Institute of Health Stroke Scale at 90 days resulted in a better outcome profile in both neurological deficit and disability [118].

### 6.6. Multiple Sclerosis (LOE = B)

A small RCT study using intravenous NAC once weekly along with 500 mg twice a day for two months showed improvement in glucose metabolism in several areas of the brain as well as improved attention and cognition in self-reported scores in the treatment group [119]. In a small study in progressive multiple sclerosis (MS), NAC at a dose of 1250 mg three times a day was well tolerated and resulted in sustained fatigue improvement [120]. Glutathione levels are reduced in secondary progressive MS and NAC is able to improve this [121].

## 7. Eye Conditions

### 7.1. Age-Related Macular Degeneration

The addition of NAC to cell cultures of retinal pigment epithelium resulted in significant reduction of oxidative damage in cell studies [122]. NAC also upregulates reduced glutathione production and also reverses lipid peroxidation in these cells [123]. This has been suggested as a novel new treatment for macular degeneration and clinical studies are warranted.

### 7.2. Glaucoma (LOE = B)

At present, there are animal studies that suggest that NAC may decrease retinal damage caused by ocular hypertension [124]. Oxidative stress and autophagy were suppressed by NAC, which increases glutathione, suggesting that this may be useful in some types of glaucoma patients [125].

### 7.3. Sjogren's Syndrome (Dry Eyes) (LOE = A)

Oral NAC used in a double-blind study has shown improvements in daytime thirst, ocular soreness, ocular irritability, and halitosis in Sjogren's syndrome [126].

## 8. Psychiatric Conditions

There may be therapeutic benefit with NAC on schizophrenia, bipolar illness, and obsessive compulsive disorder as well as other impulsive or compulsive behaviours such as seen in gambling, substance misuse, pathological nail biting, and trichotillomania [127]. A more recent review of current evidence is in agreement with the effect of NAC on various psychiatric conditions [128]. One of the mechanisms for the benefit is improvement on mitochondrial resilience against stress [12]. With regard to addiction behaviour, NAC may provide enhanced glutamate homeostasis and modulate glutamatergic dysfunction [129].

### 8.1. Schizophrenia (LOE = A)

In schizophrenia, there is glutathione dysregulation, which improves with NAC as a precursor of glutathione [130]. There is evidence in a systematic review that NAC used as adjuvant therapy improves schizophrenia symptoms and may also improve one cognitive domain in the area of working memory [131]. The improvement was seen with the longer intervention.

### 8.2. Obsessive Compulsive Disorder (LOE = A)

An RCT using NAC as an adjuvant with the use of fluoxetine showed significant improvement in the NAC group in the treatment of moderate-to-severe obsessive compulsive disorder (OCD) [132]. Another RCT using NAC as an adjuvant with the use of citalopram in children and adolescents showed significant improvement in resistance/control compulsions [133]. One systematic review of the use of NAC as an adjuvant suggests that results remain inconclusive; however, because of the relative benign side-effect profile, larger, more robust studies need to be done to determine which clinical populations would benefit from this [134]. NAC used as adjuvant in treatment of resistant OCD showed a reduction in anxiety symptoms [135].

### 8.3. Bipolar Illness (LOE = A)

A major issue in bipolar disorder is treatment-resistant subthreshold depression. An RCT (*n* = 75) using NAC as augmentation strategy was found to be safe and effective for the depressive symptoms in bipolar disorder [136].

### 8.4. Trichotillomania, Pathologic Nail Biting, and Skin Picking (LOE = A, B)

Trichotillomania has been difficult to treat, and the use of serotonergic medications has been conflicting. An RCT trial using NAC for treatment of trichotillomania showed significant benefit [137]. There are a number of case studies and short trials that show benefit in pathologic nail biting [138] and skin picking [139].

### 8.5. Addiction Behaviour (LOE = B)

In cocaine-seeking behaviour, NAC was useful in reducing relapse by providing glutamate homeostasis in animal studies [140]. In humans, reduced cravings in substance use disorders have been seen in some early studies with the use of NAC [141]. This was seen in most but not all studies in cannabis use disorder, alcohol use disorder, and smoking use disorder.

### 8.6. Use as Chelator for Metal Toxicity (LOE = A, B)

N-acetylcysteine has been shown to chelate toxic metals in animal studies as well as in human studies with little or no effect on essential metals. Mercury, lead, gold, and arsenic have been removed in humans although the studies are limited. The evidence for removal of lead is more robust because of a double-blind placebo-controlled trial [142]. Metal on metal hip prosthesis often results in increased chromium in the blood and NAC has been helpful in reducing levels safely [143]. Adverse effects of arsenic-induced hepatotoxicity in rats were countered by NAC [144]. In a case report of acute ingestion of a potentially lethal overdose of sodium arsenate ant poison, intravenous NAC reversed the clinical outcome of expected death [145].

### 8.7. Nanoparticle-Induced Reduction of Deoxyribonucleic Acid Methylation (LOE = B)

There has been increasing concern with the toxicity of nanoparticles causing cellular damage by increasing reactive oxygen species as an epigenetic mechanism decreasing deoxyribonucleic acid (DNA) methylation. Preclinical studies show that NAC reverses and prevents the oxidative damage caused by engineered nanoparticles [146].

### 8.8. Side Effects

One of the properties of NAC is that it has an unpleasant smell and taste but is generally well tolerated in oral doses below 1200 mg/day. It may also cause some nausea vomiting and diarrhea. Vomiting after intravenous use has been reported in about 11% at doses of 150 mg/kg and one anaphylactic reaction has been reported [147]. N-acetylcysteine has anticoagulant and platelet inhibiting properties and the use in patients with bleeding disorders or blood thinners may be relatively counterindicated [148]. The use of NAC with patients on nitroglycerine should be cautioned, since it may cause hypotension [149]. Other more rare side effects may include stomatitis, drowsiness, rhinorrhea, and hemoptysis [127].

### 8.9. Dosing of NAC

N-acetylcysteine dosing varies significantly with various clinical studies and doses of 1200 mg daily or more are usually required to be clinically relevant. Studies in metabolic diseases show that 5–600 mg orally per day may be sufficient to ameliorate fatty liver disease. For Crohn's disease and ulcerative colitis, doses of 800 mg per day seemed sufficient. Doses as high as 1250 mg orally three times a day have been used safely in MS and showed benefit in reducing fatigue. Doses of 8000 mg/day orally did not cause clinically significant reactions in HIV patients [150]. Clearly dosing is still debated and much needs to be learned in this area.

## 9. Discussion and Conclusion

N-acetylcysteine appears to be well tolerated with minimal side effects when used as a supplement or in treatment of various disorders. As stated above, the dosage required for this medication is not always clear, and there is much work needed to provide this information. A number of mechanisms of its actions are listed in Table 1. These actions provide reasoning for some of the results that are seen in so many different conditions. Many other conditions not listed in this document are emerging as understanding about NAC grows. As seen with other antioxidants in the past, there is some caution as expressed above with lung cancer models, where there may be an increase in proliferation as a result of p53 inhibition or likewise a possible increase in pulmonary hypertension.

As seen above, benefit has been shown with pulmonary, psychiatric, neurologic, metabolic, and infectious diseases, fertility issues, and some cancers. For most of these conditions, NAC can be used as an adjuvant, which may improve quality of life, morbidity, and mortality.

The use in metal toxicity and recent evidence in protecting DNA are also important. Much needs to be learned and more in vivo studies need to be performed to give us more confidence in using this simple compound.

## Figures and Tables

**Table 1:** N-acetylcysteine (NAC) potential mechanisms of action.

1	Action on glutathione	NAC restores glutathione (cysteine is rate limiting) [5] as seen in cell and animal studies and clinically in acetaminophen overdose.

2	Stabilizes proteins/DNA	Protects proteins by crosslinking cysteine disulfide molecules [6]. Various mechanisms of DNA repair/protection [7] as seen in animal studies and human cell studies.

3	Scavenges free radicals	Scavenging property via the redox potential of thiols [8] as demonstrated in cell culture.

4	Anti-inflammatory property	Reduces proinflammatory cytokines [9] as seen in animal studies.

5	Antioxidant property	Reduces oxidative damage [10] as seen in cell cultures.

6	Mucolytic property	Splits disulfide bonds in mucoproteins lowering viscosity [11] demonstrated in purified mucus gels and tracheal explant systems and in vitro (in a pig tracheal pouch) models.

7	Mitochondrial resilience	Neurogenesis-inducing ability [12] reduces apoptosis of mitochondria [13] as demonstrated in human dental pulp cells.

8	Metal chelation	Thiol groups provide binding sites for metals [14] in animal studies.

9	Glutamate/dopamine homeostasis	Modulates glutamate and dopamine [15] extensive studies in humans.

10	Antiviral properties	Immune modulation, anti-NF-KB properties, and other unexplored mechanisms [16] observed in vitro and in vivo.

11	Vascular endothelial growth factor	Inhibition of vascular permeability [17] as seen in human keratinocytes.

12	Adenosine triphosphate (ATP) and nitric oxide (NO) production	Increased ATP production in some cells like fibroblasts in vitro [18]. Increased nitric oxide production [19] as demonstrated in human studies.

## Data Availability

The data used to support the findings of this study are available on PubMed.
